# Cross-Linked Amylose Bio-Plastic: A Transgenic-Based Compostable Plastic Alternative

**DOI:** 10.3390/ijms18102075

**Published:** 2017-09-30

**Authors:** Domenico Sagnelli, Kourosh Hooshmand, Gerdi Christine Kemmer, Jacob J. K. Kirkensgaard, Kell Mortensen, Concetta Valeria L. Giosafatto, Mette Holse, Kim H. Hebelstrup, Jinsong Bao, Wolfgang Stelte, Anne-Belinda Bjerre, Andreas Blennow

**Affiliations:** 1 Department of Plant and Environmental Sciences, University of Copenhagen, 1871 Frederiksberg, Denmark; kourosh.hooshmand@agro.au.dk (K.H.); gzq355@alumni.ku.dk (G.C.K.); abl@plen.ku.dk (A.B.); 2 Niels Bohr Institute, University of Copenhagen, 2100 Copenhagen, Denmark; jjkk@nbi.ku.dk (J.J.K.K.); kell@nbi.ku.dk (K.M.); 3 Department of Chemical Science, University of Naples, 80126 Napoli, Italy; giosafat@unina.it; 4 Department of Food Science, University of Copenhagen, 1958 Frederiksberg, Denmark; metteholse@hotmail.com; 5 Department of Molecular Biology and Genetics, Aarhus University, 4200 Slagelse, Denmark; kim.hebelstrup@mbg.au.dk; 6 College of Agriculture and Biotechnology, Zhejiang University, Hangzhou 310029, China; jsbao@zju.edu.cn; 7 Center for Bioresources and Biorefinery, Danish Technological Institute, Gregersenvej 7, 2630 Taatsrup, Denmark; wst@teknologisk.dk (W.S.); anbj@teknologisk.dk (A.-B.B.)

**Keywords:** starch, amylose, bioplastic, cross-linker, amylose permeability, cross-link assay, citric acid

## Abstract

Bio-plastics and bio-materials are composed of natural or biomass derived polymers, offering solutions to solve immediate environmental issues. Polysaccharide-based bio-plastics represent important alternatives to conventional plastic because of their intrinsic biodegradable nature. Amylose-only (AO), an engineered barley starch with 99% amylose, was tested to produce cross-linked all-natural bioplastic using normal barley starch as a control. Glycerol was used as plasticizer and citrate cross-linking was used to improve the mechanical properties of cross-linked AO starch extrudates. Extrusion converted the control starch from A-type to Vh- and B-type crystals, showing a complete melting of the starch crystals in the raw starch granules. The cross-linked AO and control starch specimens displayed an additional wide-angle diffraction reflection. Phospholipids complexed with Vh-type single helices constituted an integrated part of the AO starch specimens. Gas permeability tests of selected starch-based prototypes demonstrated properties comparable to that of commercial Mater-Bi^©^ plastic. The cross-linked AO prototypes had composting characteristics not different from the control, indicating that the modified starch behaves the same as normal starch. The data shows the feasibility of producing all-natural bioplastic using designer starch as raw material.

## 1. Introduction

In March 2013, the European Commission published a green paper that outlined a strategy for decreasing the impact of plastic on the environment. The primary goal of the new strategy was to promote bio-based and biodegradable plastics [1]. Bio-plastics offer great opportunities for smart and green societal growth [2].

Plastics are materials composed of natural or synthetic high molecular weight polymeric molecules. These can be shaped and used in place of other materials like glass, wood, and metals. Their properties are adaptable to many applications using additives or alternative processing technologies. Bio-plastic is a sub-group that contains two qualitative characteristics: it is bio-based and/or biodegradable. The term “bio-based” refers to materials derived from biomass, whereas biodegradable refers to materials that can be assimilated by microorganisms. The main environmental threat of conventional plastics is their low degradability rates and the persistence of macro- and micro-plastics in both soil and water [3]. Micro-plastics are classified as 1–5 mm-sized particles [4]. Over the last years, thermoplastic polymer production has increased, leading to an extensive accumulation of micro-plastics in the environment, and becoming an increasing threat to marine organisms. These particles can carry pesticides. Moreover, their ingestion by sea animals introduces these pollutants to the food chain [5].

As opposed to most conventional plastics, biodegradable plastics are metabolized by environmental microorganisms, and therefore plant-based bio-plastics represent one of the most interesting clean alternative to conventional plastics. Their biochemical structure, favorable physiochemical assets, abundance in nature, and well-established fabrication technologies for plastics support development of novel bio-plastics types [6,7,8,9], offering two major, distinct advantages, mainly being that of their renewability and availability. Starch especially, is a raw material suitable for production of new plant-based bio-plastics (e.g., Mater-Bi^©^, Novamont) due to its abundancy, low cost, and processability with the use of existing technologies.

Starch is heterogeneous and its composition typically entails two main polysaccharide types; amylopectin, a branched polymer, and amylose, a chiefly linear polymer. Amylose forms single and double helices that can align in ordered structures. The single helix provides a large hydrophobic cavity that can accommodate hydrophobic molecules, such as lipids [10,11,12,13,14,15], and lipid content is positively correlated to the amount of amylose in the granule [16]. The natural presence of lipids in the amylose helices may have appealing characteristics for materials science, but their effects on material functionality are not well characterized. Thermoplastic starch (TPS) is an edible and compostable product with functionality that can replace many conventional plastics, thereby assisting the reduction of non-degradable residue release into the environment. The production volume and technical knowledge for different TPS types is steadily increasing, and TPS can now be adapted to a variety of applications. However, it still presents certain limitations due to aging, and brittleness due to recrystallization. As an effect, starch is chemically modified to improve its stability and widen its usability. Etherification, esterification, or oxidation of the available hydroxyl groups on the glucose units are among the most commonly used approaches. For example, hydroxyethylated starch (e.g., vinyl-starch) is grafted with reagents such as 1,3-butadiene and styrene to improve coating properties in paper industries. Cross-linking is a common tactic to improve starch bio-plastic mechanical performances. Improvement of the mechanical properties of starch materials have been achieved with reagents such as sodium trimetaphosphate, phosphorus oxychloride, epichlorohydrin, sodium tripolyphosphate, and 1,2,3,4-diepoxybutane. Most of these compounds are unsafe and potentially toxic, and the use of citric acid in conjunction with sodium hypophosphite is considered to be a cleaner cross-linking technology [17].

Polymer industry and research are increasingly pointing towards more environment-friendly solutions for plastics production. Recent investigations pinpoint opportunities of plant-based biomaterials and composites as food packaging for short- and long shelf-life products [18]. If produced by all-natural plant-based raw materials [9], such products will have a tremendous impact on the economy and environment.

An attractive approach is the direct modification of polysaccharides in crops using transgenic techniques. One example is amylose-only (AO) starch, produced by a transgenic barley line synthesizing a starch having 99% amylose. This starch was generated by RNA interference suppressing all three starch branching enzymes in the barley. The AO starch, as shown previously [6], provides a useful raw material for bio-plastics fabrication. In the present study, we extended this initiative using a scale-up extruder and investigating cross-linking with citric acid (CA) with the aim to produce a more robust bio-sustainable, yet compostable bio-plastic. Prototypes were characterized for crystallinity, mechanics, gas permeability, and composting biodegradation.

## 2. Results and Discussion

### 2.1. Determination of Lipid Content

The presence of lipids complexed to amylose is expected to have a large influence on starch physics. The major internal lipids found for the AO and the control starch samples were phospholipids, especially lysophosphatidylcholine (lysoPC), as showed by TLC analysis visualized using primuline dye (Figure S1).

AO showed a 40-fold higher free fatty acid content as compared to the control starch (Table 1). These data demonstrate that the increased content of amylose in the starch granule is positively correlated to the increase of lipids.

### 2.2. Monitoring of Cross-Linking Reaction

**Figure 1:**
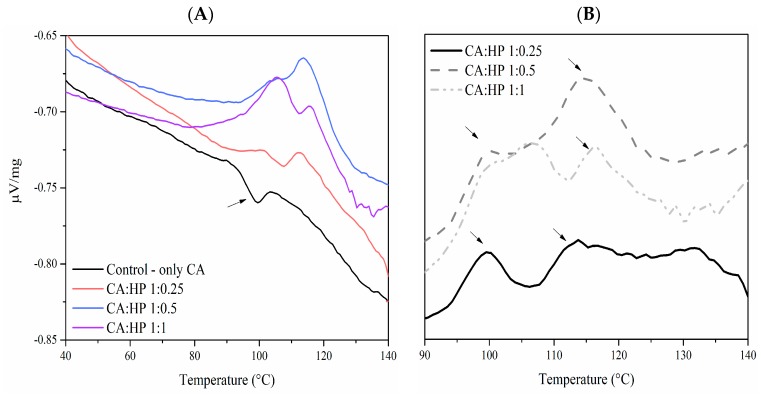
(**A**) Differential scanning calorimetry (DSC) thermograms of starches showing exergonic citric acid (CA) cross-linking reactions in a sodium hypophosphite (HP)-dependent manner. The arrow indicates an endergonic reaction; (**B**) Control data subtracted indicating HP-dependent cross-linking exotherms (arrows).

To identify optimal cross-linking conditions, the CA cross-linking reaction temperature was monitored using differential scanning calorimetry (DSC) before the extrusion, with or without sodium hypophosphite (HP) catalyst included. In the absence of a HP catalyst, no exergonic transition was detected (Figure 1A) indicating the absence or presence of only minor cross-linking. The concentration of catalyst needed for the cross-linking was optimized using three CA:HP ratios (CA:HP; *w:w*; 1:1; 1:0.5; 1:0.25). All three conditions showed exergonic transitions with increasing energy release proportional to the amount of HP catalyst used (Figure 1A). Subtracting the control (without HP) thermogram to the CA/HP thermograms, two HP-dependent exergonic peaks could be identified (Figure 1B). The first occurred at 100–105 °C and the second at 120–125 °C. The extrusion protocol was designed with these catalytic events under consideration. In order to avoid a high amount of residual catalyst in the final extrudate, the ratio CA/HP used for the extrusion was set at low level, 1:0.25.

**Figure 2:**
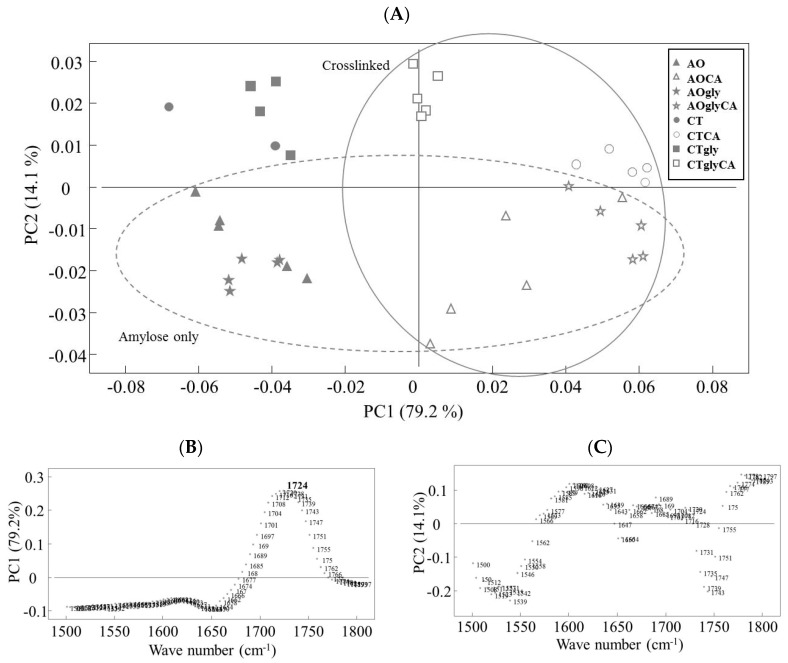
Principal component analysis (PCA) on Multiplicative Scatter Correction pre-processed Fourier Transform Infrared Spectroscopy spectra (1800–1500 cm^−1^) obtained from analysis of extrudates. (**A**) Score plot of PC1 vs. PC2. Samples are grouped according to both starch type and CA a cross-linking; (**B**,**C**) loading plots of PC1 and PC2, respectively. The peak arising from carboxyl and ester carbonyl absorbance (1724 cm^−1^) is highlighted.

To confirm the formation of ester bonds, Fourier Transform Infrared Spectroscopy (FTIR) analysis was performed with data being collected from 4000–550 cm^−1^ with a spectral resolution of 8 cm^−1^ and attention focused to 1724 cm^−1^ (Figure S2), previously ascribed to the carboxyl and ester carbonyl bands [17]. From the loadings and scores of the principal component analysis (PCA) model, which clearly discriminated the samples treated with CA, it was concluded that the extrusion conditions were appropriate (Figure 2).

### 2.3. Crystalline Properties of the Extrudates

The extrudates were characterized for crystallinity by wide angle X-ray scattering (WAXS), mechanical analysis, and gas permeability. After the melting process and prior to any analysis, the extrudates were stored in a controlled environment stabilizing the semi-crystalline matrix.

**Figure 3:**
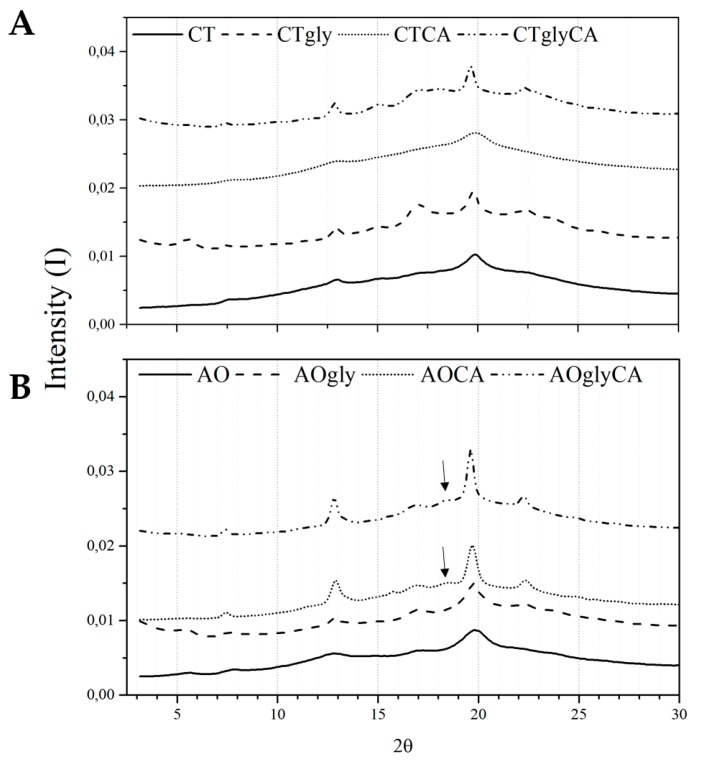
Wide angle X-ray scattering (WAXS) diffractograms of extruded samples showing the effect of glycerol and CA cross-linking on crystal formation: (**A**) Control starch; (**B**) AO starch. Arrows highlight the A-type polymorph.

For WAXS analysis, the extrudates were stored at 85% relative humidity (RH). The effect of both cross-linking and glycerol was evident, with both treatments increasing the crystallinity of the extrudates (Figure 3). Previously, we demonstrated that native AO starch and extrudates fabricated from this starch only encompassed Vh- and B-type crystallinity [6]. Cross-linking of both AO and control induced the formation of a mixture of Vh, B-, and a further crystal polymorph that may be associated to A-type [18]. The Vh-type polymorph was substantiated by reflexions at 2θ 7.3, 12.8, 19.6, the A-type at 2θ 15, 18.8 and the B-type at 2θ 5.5, 17.1, 22.3 (Figure 3). Both CA cross-linking and glycerol facilitated the growth of all crystalline polymorphs. Particularly, the presence of the additives resulted in sharper peaks. The samples without plasticizer and cross-linker were almost entirely amorphous. An exception was represented by the control starch treated only with CA that showed lower crystallinity than AO/CA.

### 2.4. Mechanical Properties and Glass Transition

**Figure 4:**
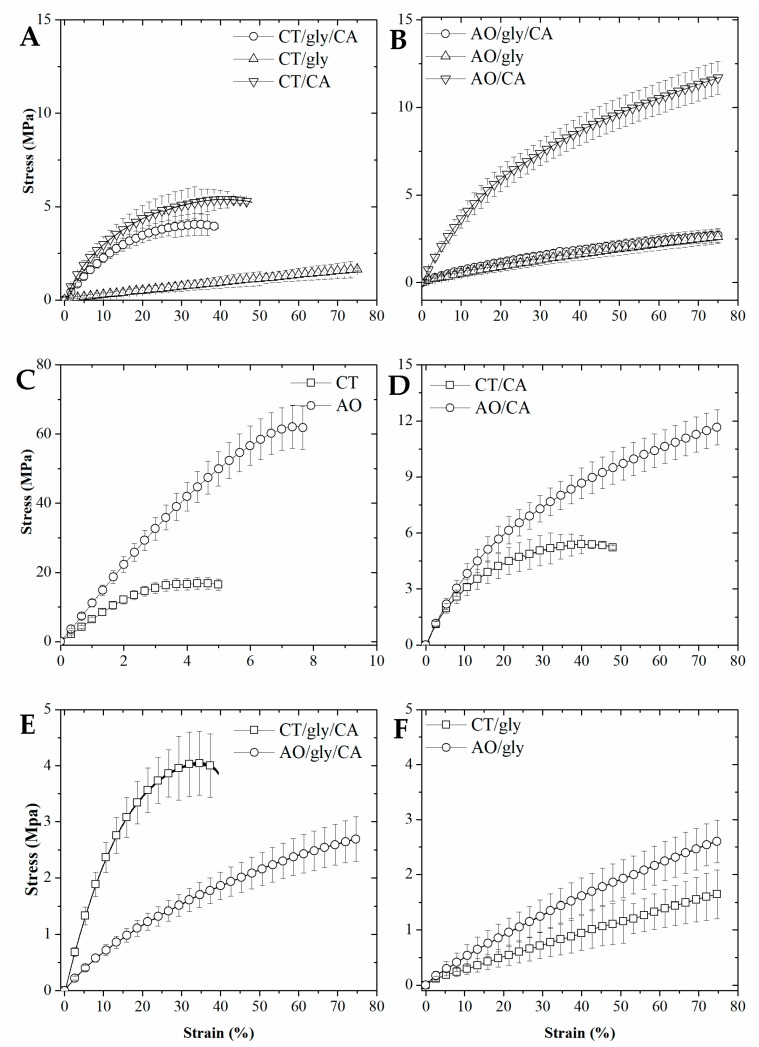
The stress and strain of extruded samples. (**A**) Control starch extrudates; (**B**) AO starch extrudates; (**C**) Comparison of pure starch (no glycerol) extrudates; (**D**) Comparison of extrudates cross-linked with CA; (**E**) Comparison of extrudates cross-linked and with glycerol; (**F**) Comparison of extrudates with glycerol. Error bar represent the standard deviation.

The extruded specimens, after equilibration at 57% RH, were tested by tensile tests to characterize their stress and strain behavior in standard conditions. The utilization of a double barrel extruder and controlled water feeding allowed the production of pure starch extrudates. The AO-extrudates had superior performance compared to the control starch extrudates, with AO having five-fold higher stress at break and 1.6-fold higher strain at break as compared to the control starch specimens (Figure 4A–C). Lack of glycerol plasticizer made the specimens glassy and brittle. Cross-linking had a noteworthy effect on the extrudates, which become more flexible and cohesive even without glycerol as a plasticizer (Figure 4D). CA cross-linking increased strain at break for both AO and control starch specimens. The AO extrudates with only cross-linker (AOCA) samples showed a strain at break that was 1.6-fold higher, and a stress at break that was two-fold higher than the control (Figure 4D). In fact, CA cross-linking resulted in a better plasticizing effect than glycerol, as showed by comparing the stress and strain curves of AO with only glycerol and the cross-linked samples without glycerol. The glycerol plasticized samples had higher elasticity but lower strength as compared to the cross-linked ones. (Figure 4D,F). The AO-based specimens including glycerol with CA crosslinking (AOglyCA) extrudates had higher elasticity but a lower strength as compared to the control (Figure 4E). However, for AO the use of only glycerol and the glycerol/CA combination did not differ significantly (Figure 4B). The effect of the cross-linker on the glass transition temperature (Tg) of AO extrudates was monitored by using dynamic mechanical analysis (DMA). The AO-based specimens including glycerol, AOgly, had a Tg = 50 ± 1 °C. Introducing CA cross-linking to this system (AOglyCA) decreased the Tg to 38 ± 3 °C. AOCA had a Tg that was much higher than all other samples (80 ± 2 °C), indicating that CA formed a densely cross-linked network with the AO chains. This network was very sensitive to glycerol as demonstrated by the very low Tg of the AOglyCA specimens.

### 2.5. Permeability Tests

**Figure 5:**
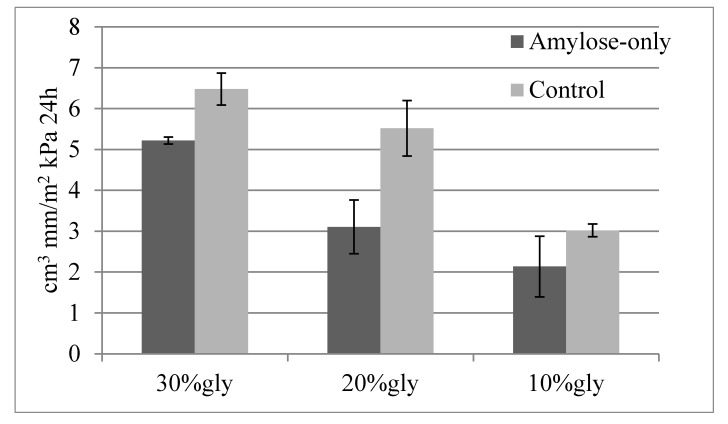
Permeability tested for non-crosslinked AO and control starch films as a function of glycerol content.

The permeability tests for cast AO and control films showed performances similar to those of commercial plastics (Mater-Bi^©^ and Low-Density Poly-Ethylene, LD-PE) (Table 2). The permeability to CO_2_ and O_2_ of the starches analyzed was comparable to that of the commercial variants. Mater-Bi was superior regarding the water vapor permeability (WVP) permeability. Hence, the gravimetric tests showed a significant effect of glycerol on water vapor permeability. The permeability increased by increasing the amount of glycerol in the formulation (Figure 5).

This data supports the high potential behind the use of starch for the production of plastics.

### 2.6. Biodegradation of Grains and Extruded Prototypes

**Figure 6:**
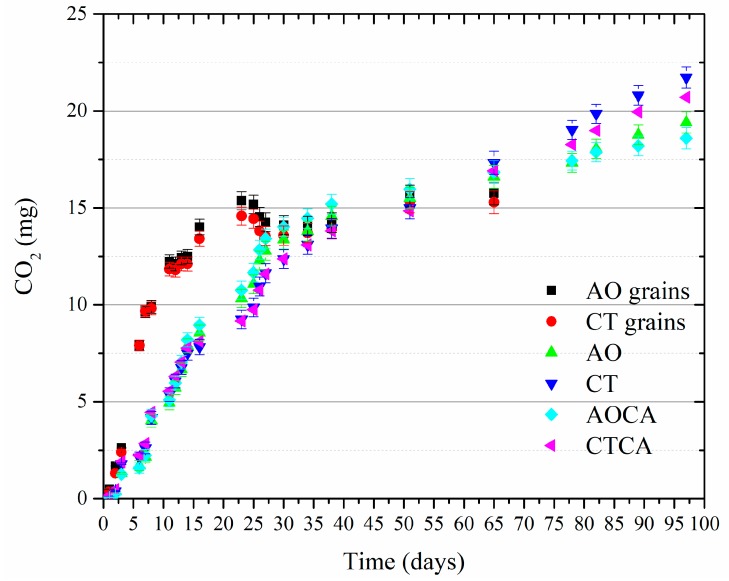
CO_2_ release during the biodegradation test of AO and control grain and bioplastics protopypes. The data has been subtracted from the soil reference data.

Controlled biodegradation in a composting system of milled AO and control barley grains, and extruded specimens from starch prepared from these, was monitored as CO_2_ evolution (Figure 6, Tables S1 and S2). The grain and the extruded samples showed significant differences; the milled grain was degraded faster than the bio-plastics prototypes. No lag phase in the beginning of the composting was observed for any of the samples demonstrating high biological activity in the soil. No significant difference was found between the AO and the control grain. Likewise, the AO and the control starch bio-plastic prototypes showed no significant difference, and there was also no significant difference between cross-linked and non-cross-linked samples (Figure 6). The slower degradation of the extruded samples as compared to the milled grain was most likely due to the compact structure of the extrudates. Enzymatic degradation of solid substrates is a surface phenomenon, and therefore strongly affected by the accessibility of the enzymes to the substrate and the availability of moisture. The background CO_2_ evolutions of organic matter present in the soil were similar in both tests. The rate of degradation was highest during the first 20 days, and decreasing significantly afterward. After 100 days, the degradation had the same rates as the soil reference concluding that all test specimen were fully degraded at that stage. The results demonstrates that transgenic thermoplastic AO starch is not significantly different with respect to composting, as compared to a common thermoplastic starch. 

## 3. Materials and Method

### 3.1. Materials

Barley starch used in this study was extracted and purified from two barley lines: a control barley and amylose-only, a genetically modified line. The AO line was generated by RNA interference suppressing all three starch branching enzymes in the Golden Promise cultivar background [20]. All chemicals used were provided by Sigma-Aldrich (St. Louis, MO, USA). For composting trials, topsoil was collected from fallow land located in the municipality of Taastrup, Denmark (N 55°39′33″; E 12°16′6″).

### 3.2. Methods

#### 3.2.1. Determination of Lipids

The lipid extraction was performed as described by Morrison and coworkers [21]. Butanol extracts were applied on a thin layer chromatography plate (TLC silica gel 60, Merck Darmstadt, Germany) together with appropriate standards (PC: Phosphatidylcholine, PG: Phosphatidylglycerol, PE: Phosphatidylethanolamine, PS: Phosphatidylserine, lysoPC: Lysophosphatidylcholine, and oleic acid (free fatty acid)). The TLC development was performed using chloroform/ethanol/water/triethylamine (30/35/7/35; *v/v/v/v*). The TLC plates were assessed using a Typhoon Trio variable-mode imager (GE Healthcare, Brøndby, Denmark). Lipid extracts from starch were analyzed using a phosphorous assay [22]. Shortly, the extracts together with a standard phosphate solution (50 to 200 nmol) were digested by incubation in 0.65 mL 72% perchloric acid at 195 °C until colorless. Afterward, 3.3 mL of water, 0.5 mL of 2.5% ammonium molybdate, and 0.5 mL of 10% ascorbic acid (*w/v*) were added to the solution. The color was developed incubating the samples in a water bath at a temperature of 80 °C for 10 min. Samples were rapidly cooled on ice water bath and the absorbance was measured at 812 nm using a GENESYS 10 spectrophotometer (Thermo Electron Corporation, Madison, WI, USA).

#### 3.2.2. Cross-Linking Evaluation by Differential Scanning Calorimetry

The cross-linking reaction was performed using CA and HP as a catalyst. During the heating process (described below), the CA is converted to an acid anhydride, which reacts with the hydroxyl groups of the starch forming ester linkages. The cross-linking temperature was determined by DSC using a DSC 214 polyma (Netzsch, Selb, Germany) instrument. The experiments were carried out on aliquots of 20 ± 1 mg of the polymer, which was placed in stainless steel air tight cells. A single scan was performed from 30 to 140 °C by an incremental increase of temperature of 3 °C·min^−1^.

#### 3.2.3. Melt Processing

The extrusion process was performed on a laboratory scale co-rotating inter-meshing twin-screw extruder (Process 11, Thermo Fisher Scientific, Karlsruhe, Germany). The barrel diameter and its length-to-diameter ratio (L/D) were 11 mm and 40:1, respectively. The extruder barrel was fitted with a circular 3 mm die nozzle. The extruder was powered by a 1.5 kW motor and the screw speed was kept constant at 350 rpm. The extruder had seven internal and one external heating zones and the temperature profile was optimized and set as shown in Table 3. A high shear screw configuration was applied. The screw configuration was designed to produce homogeneity in the formulations. Permanence of the formulation in the heating block was permitted by an inverted screw positioned at the end of the sixth heating block (115 °C). The raw material was metered into the extruder by a gravimetric twin-screw feeder (MT-S, MiniTwin, Brabender Technologie, Duisburg, Germany) at a speed of 0.8 kg/h. Water, or water plus cross-linker was pumped into the extruder using a peristaltic pump (Fillmaster Type 421, Delta Scientific Medical, Store Heddinge, Denmark) at a speed of 6 mL/min resulting in a final ratio of 1:0.5 with the starch (dry weight, d.w.). Glycerol was pumped into the extruder using a second peristaltic pump (Gilson Inc., Minipulse 3) at a speed of 3 mL/min resulting in a final ratio of 1:0.33 with the starch (d.w.). Four formulations were prepared per starch type consisting of starch mixed with water alone, water plus glycerol, water plus cross-linker, and water plus cross-linker plus glycerol. The dry starch:CA ratio was 1:0.25, and all blends were prepared by taking into account the dry weight of starch.

The nomenclature of the samples is indicated accordingly: starch type/plasticizer/cross-linker where the starch types were AO (amylose-only) or CT (control), the plasticizer gly (glycerol) and CA cross-linker. For example, for a plasticized cross-linked AO the nomenclature is: AOglyCA.

#### 3.2.4. Fourier Transform Infrared Spectroscopy

The absorbance measurements were performed on an Arid-Zone MB100 FTIR instrument (ABB Bomen, QC, Canada) equipped with an attenuated total reflectance (ATR) device with a triple-bounce diamond crystal. IR spectra were recorded in the range from 4000–550 cm^−1^ with a spectral resolution of 8 cm^−1^. The milled sample was squeezed against the crystal surface with a concave needle compressor. Each spectrum represents the average of 32 scans ratio against the background (64 scans measured on the surrounding air) and analysed in triplicate. Data analysis (pre-processing, principal component analysis—PCA) was performed using LatentiX (v.2.12) in the range 1500–1800 cm^−1^.

#### 3.2.5. Wide-Angle X-ray Scattering

WAXS of the hydrated samples was performed using the SAXSLab instrument, NBI, University of Copenhagen, equipped with a 100XL + micro-focus sealed X-ray tube (Rigaku) with a 1.54 Å beam. The water content of the samples was adjusted by water phase sorption for 14 days in desiccators at a relative humidity of 85% (saturated KCl) [18].

#### 3.2.6. Mechanical Properties

Prior to any measurements, the samples were equilibrated in a desiccator at 57% ± 1 relative humidity (saturated NaBr). The specimens were analyzed using a texture analyzer (TA, Stable Micro Systems, Surrey, UK) equipped with a 300 N tensile load cell. The distance between the clamps was set at 10 mm and the crosshead speed was set at 10 mm min^−1^. The elongation and the tensile stress at break were measured at 25 °C in triplicate.

#### 3.2.7. Dynamic Mechanical Analysis

A DMA Q800 (TA Instruments, New Castle, DE, USA) was used in cantilever mode with an amplitude of 0.1% at a frequency of 10 Hz, standard heating rate of 3 °C·min^−1^ and a ramp between −60 to 150 °C. The experiments were carried out on prototypes with a length of 4 cm and an average width of 5 ± 0.1 mm.

#### 3.2.8. Permeability to Gases

Gas permeability was monitored using thin films prepared by casting. A 2% (*w/w*) starch suspension was gelatinized using a microwave oven for 3 min in a Duran bottle closed using a membrane screw-cap to avoid over-pressure. After gelatinization, the samples were stirred for 5 min at 300 rpm and poured in the petri dishes coated with teflon, and preheated at 70 °C at a surface density of 20 mg/cm^2^. The suspensions were dried at 70 °C for 3 h using maximum oven ventilation and there after at 50 °C for 10 to 12 h without ventilation. CO_2_, O_2_, and H_2_O permeability were determined using the American Society for Testing and Materials (ASTM) Standard Method D 3985 (2010) and F1249 (MultiPerm apparatus-ExtraSolution s.r.l., Pisa, Italy). Duplicate samples were conditioned for 2 days at 50% RH before the measurement. Aluminum masks were used to reduce film test area to 5 cm^2^. The testing was performed at 25 °C and 50% RH.

The effect of glycerol plasticizer on the water vapor permeability was performed by a gravimetric test according to ASTM E96 using in-house designed permeability cups in triplicate. The experiments were performed in a desiccator with an RH of 84% using a saturated solution of KCl. The samples were weighed every 30 min the first day (totally 16 measurements) and then every h for 8 h on the following days for at least three days (eight measurements/day).

#### 3.2.9. Biodegradation Test

The biodegradation test was slightly modified from the ASTM standard [23]. Large size impurities such as stones and organic materials i.e. leaves, roots were removed from the soil using a 10-mm metal screen. The soil was subsequently screened to a particle size of <2 mm using a sieving tower with standardized metal screens and a vibrating plate (Retsch, Haan, Germany) (Figures S3–S5). pH was determined in duplicate according to the working document “Determination of pH in soil, sewage sludge and bio-waste” STD5151 [24]. The extruded, bio-plastic prototypes were cut into 5 mm long tube-shaped pieces. Bio-plastic grain samples were separated from fines using a sieving tower and screens with 2 mm mesh size. 2 g of each material was buried in the soil at 5–10 mm below the surface. For CO_2_ capture and maintenance of a moist atmosphere, a set of beakers containing 20 ml de-mineralized water and 20 mL of KOH (0.5 N) were placed on the perforated plate inside the desiccators. These were sealed and placed in a dark chamber in a climate controlled room at a temperature of 20 ± 1 °C. The desiccators were ventilated at regular intervals, and the CO_2_ traps were renewed on a regular basis following the standard procedure. Evolved CO_2_, absorbed into the KOH solutions was determined by titrating triplicates with HCl (0.5 N) to a phenolphthalein end-point.

## 4. Conclusions

Massive thermoplastic polymer production is leading to an extensive accumulation of micro-plastics in the environment, and is becoming an increasing environmental threat. All-natural, biodegradable bioplastics can form part of this solution, but performance must be improved. We produced and tested extruded and CA cross-linked specimens prepared from AO barley starch. This material had superior mechanical performance as compared to specimens from normal barley starch. Cross-linking produced an additional crystal related to the A-type polymorph. CA cross-linking improved mechanical strength and the cross-linked AO extrudates had superior stress at break and elongation at break. Glycerol plasticizer decreased its performance, and despite its high elasticity, the Tg of the cross-linked AO extrudates was increased indicating the presence of a strongly cross-linked network. AO contained amylose-complexed phospholipids, with free fatty acids constituting an integrated part of the Vh crystals in the specimens. Permeability to CO_2_, O_2_, and H_2_O was comparable to commercial starch-based blends. Biodegradation in composting systems demonstrated that AO designer starch and cross-linked starch degraded at the same rate as normal starch. This new material showed that bioplastic produced using a polysaccharide from a genetically modified plant could be a functional alternative to specific flexible plastics and opens new possibilities for the production of biodegradable materials from e.g., polysaccharides, produced directly in planta, having properties specifically tailored for bioplastics applications.

## Figures and Tables

**Table 1:** Free fatty acids (FFA) and phospholipids (PPH) in amylose-only (AO) and control.

Sample	FFA (%)	PPH (%)
Amylose-only	0.04	0.9
Control	0.001	0.5

**Table 2:** Gas permeability of AO-based films compared to selected commercially available materials.

Permebility	CO_2_ (cm^3^·mm/m^2^·kPa 24 h)	O_2_ (cm^3^·mm/m^2^·kPa 24 h)	WVP (cm^3^·mm/m^2^·kPa 24 h)
AO/gly	4.0 ± 0.2	0.6 ± 0.03	0.1 ± 0.01
AO/gly/CA	0.4 ± 0.1	1	0.1 ± 0.03
CT/gly	NA ^$^	NA ^$^	0.1
CT/gly/CA	0.5	0.5	0.02
Mater-Bi (S-301)	5.0 ± 0.03	0.7 ± 0.005	0.04 ± 0.002
Mater-Bi ZIO1U/C	5.0 ± 0.02	0.5 ± 0.003	Na
Mater-Bi (Z)	Na	Na	33 *^a^*
LD-PE	Na	Na	0.5 *^a^*

Na: not available *^a^* data published in Mariniello et al. 2007 [19], ^$^ the film was too fragile for the analysis. water vapor permeability (WVP).

**Table 3:** Screw configuration and temperature profile. The screws were organized to allow longer permanence of starch into the cross-linking heating blocks. The numbers represents the heating and feeding blocks configuration temperatures.

	Cross-Linking				Glycerol	Water/CA	Starch
135 °C	125 °C	115 °C	115 °C	105 °C	80 °C	40 °C	40 °C
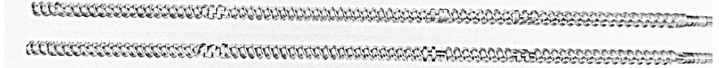							

## Supplementary Materials

Supplementary materials can be found at www.mdpi.com/1422-0067/18/10/2075/s1.
