# Supplementary material for: Cross-Linked Amylose Bio-Plastic: A Transgenic-Based Compostable Plastic Alternative
Source: Int J Mol Sci. 2017 Sep 30;18(10):2075. doi: 10.3390/ijms18102075 (PMC5666757; doi:10.3390/ijms18102075)
Supplement: Supplementary file 1 [file ijms-18-02075-s001.pdf]

Cross-Linked Amylose Bio-Plastic: A Transgenic-Based Compostable Plastic Alternative

Domenico Sagnelli [1,*], Kourosh Hooshmand [1], Gerdi Christine Kemmer [1], Jacob J.K. Kirkensgaard [2], Kell Mortensen [2], Valeria Giosafatto [3], Mette Holse [4], Kim H. Hebelstrup [5], Jinsong Bao [6], Wolfgang Stelte [7], Anne-Belinda Bjerre [7], Andreas Blennow [1]

[1] Department of Plant and Environmental Sciences, University of Copenhagen, Denmark; kourosh.hooshmand@agro.au.dk (K.H.); gzq355@alumni.ku.dk (G.C.K.); abl@plen.ku.dk (A.B.)
[2] Niels Bohr Institute, University of Copenhagen, Denmark; jjkk@nbi.ku.dk (J.J.K.K.); kell@nbi.ku.dk (K.M.)
[3] Department of Chemical Science, University of Naples, Italy; giosafat@unina.it;
[4] Department of Food Science, University of Copenhagen, Denmark; metteholse@hotmail.com
[5] Department of Molecular Biology and Genetics, University of Aarhus, Denmark; kim.hebelstrup@mbg.au.dk
[6] Zhejiang University, Hangzhou, China; jsbao@zju.edu.cn
[7] Department of climate and energy, Danish technological institute, Denmark; wst@teknologisk.dk (W.S.); anbj@teknologisk.dk (A.-B.B.)
* Correspondence: dsagnelli@plen.ku.dk

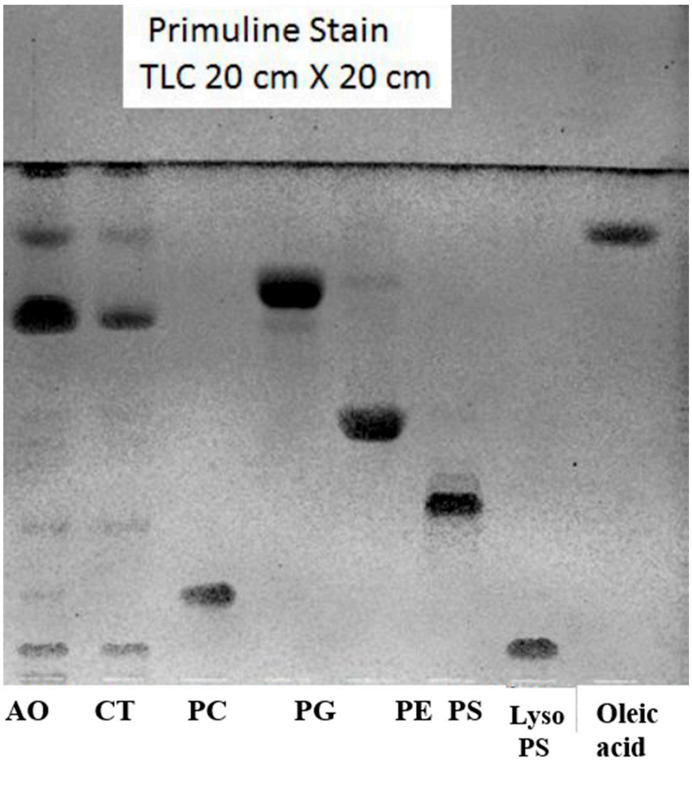

Supplementary Figure 1. Phopholipids determination. AO: amylose-only, CT: control (*Hordeum vulgare*) Standards: PC: Phosphatidylcholine, PG: Phosphatidylglycerol, PE: Phosphatidylethanolamine, PS: Phosphatidylserine, lysoPC: Lysophosphatidylcholine, and oleic acid (free fatty acid).

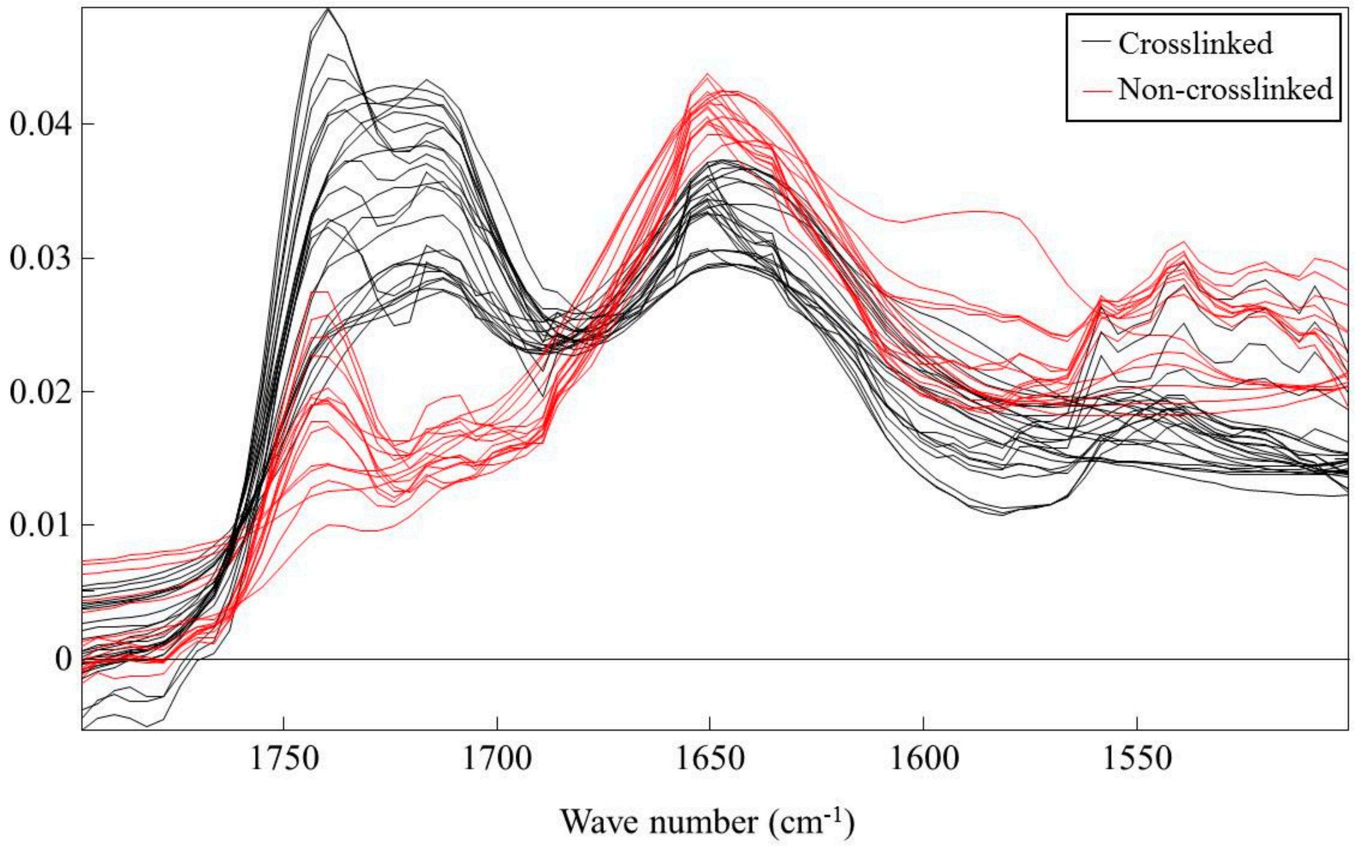

Supplementary Figure 2: MSC pre-processed FTIR spectra (1800-1500cm^{-1}) obtained from analysis of the extrudates.

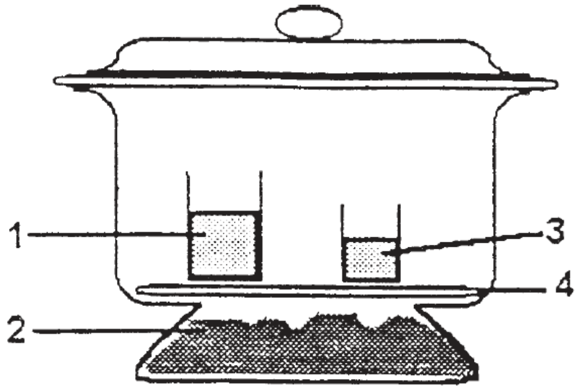

Supplementary Figure 3. Soil incubator with 1) potassium hydroxide solution, (2) soil, (3) water, and (4) perforated plate. Adapted from *ASTM-D5988-03, 2012*

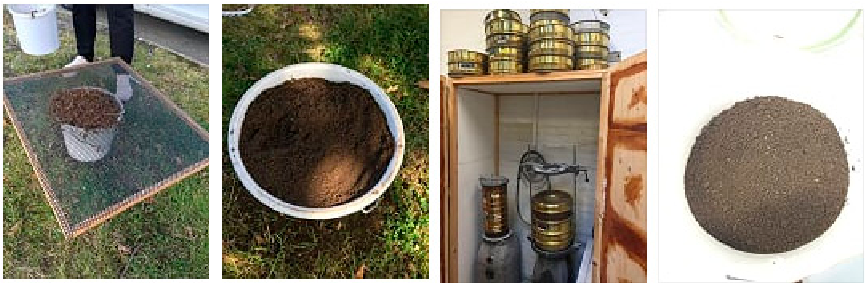

Supplementary Figure 4. Preparation of soil samples. Screening in a 10 mm mesh and a sieving tower with 2 mm screen.

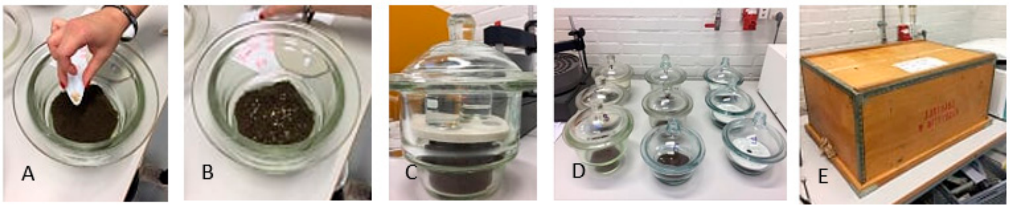

Supplementary Figure 5. A, B: Sample is spread out and covered with soil. E, D: Beakers with KOH (0.5 N) and water are added on top and the desiccators are sealed and placed into the darkened chamber in a climate controlled laboratory.

Supplementary table 1. CO_2 release during the biodegradation test of High amylose barley grains and reference barley grains.

**Time [days]**	**Evolved CO_2 High amylose barley grains [ml]**			**Evolved CO_2 Reference barley grains [ml]**		
1	1,24	±	c	1,11	±	0,00
2	2,89	±	0,02	2,52	±	0,02
3	4,82	±	0,04	4,60	±	0,04
6	10,44	±	0,27	10,45	±	0,11
7	12,83	±	0,30	12,86	±	0,14
8	14,47	±	0,33	14,39	±	0,20
11	17,62	±	0,35	17,23	±	0,31
12	18,26	±	0,35	17,94	±	0,32
13	19,01	±	0,35	18,70	±	0,32
14	19,65	±	0,36	19,26	±	0,33
16	21,67	±	0,40	21,07	±	0,35
23	24,31	±	0,47	23,52	±	0,40
25	25,14	±	0,48	24,39	±	0,41
26	25,46	±	0,49	24,75	±	0,41
27	25,70	±	0,49	24,99	±	0,42
30	26,01	±	0,49	25,47	±	0,42
34	26,72	±	0,50	26,36	±	0,44
38	27,28	±	0,51	26,96	±	0,46
51	29,06	±	0,55	28,73	±	0,47
65	30,49	±	0,59	30,04	±	0,50

Supplementary table 2. CO_2 release during the biodegradation test of bioplastics.

**Time [days]**	**Evolved CO2 High amylose starch [ml]**			**Evolved CO2 High amylose starch crosslinked [ml]**			**Evolved CO2 Reference [ml]**			**Evolved CO2 Reference crosslinked [ml]**		
1	0,83	±	0,83	0,83	±	0,02	0,82	±	0,820	0,92	±	0,02
2	1,58	±	0,83	1,60	±	0,04	1,45	±	0,824	1,68	±	0,05
5	3,51	±	0,84	3,99	±	0,05	3,46	±	0,832	4,09	±	0,06
6	4,25	±	0,85	4,67	±	0,07	4,13	±	0,842	4,78	±	0,08
8	5,35	±	0,88	5,81	±	0,10	5,43	±	0,855	6,03	±	0,10
13	8,60	±	0,90	8,74	±	0,11	8,85	±	0,908	9,03	±	0,11
15	10,33	±	0,90	10,75	±	0,11	10,48	±	0,914	10,93	±	0,12
18	11,88	±	0,91	12,22	±	0,15	12,12	±	0,933	12,46	±	0,16
21	13,25	±	0,92	13,35	±	0,17	13,63	±	0,946	13,64	±	0,17
25	15,04	±	0,94	14,65	±	0,20	15,35	±	0,983	14,92	±	0,19
27	16,21	±	0,95	15,48	±	0,20	16,61	±	1,012	15,77	±	0,21
32	19,27	±	0,95	18,19	±	0,22	19,70	±	1,021	18,11	±	0,26
35	21,02	±	0,96	19,82	±	0,24	21,61	±	1,030	19,70	±	0,30
40	23,22	±	0,98	21,87	±	0,25	23,76	±	1,033	21,68	±	0,31
44	24,23	±	0,98	23,08	±	0,25	24,86	±	1,039	23,04	±	0,32
47	25,26	±	0,99	24,26	±	0,26	25,92	±	1,043	24,27	±	0,32
51	26,44	±	0,99	25,76	±	0,30	27,11	±	1,054	25,74	±	0,33
54	27,62	±	1,02	26,99	±	0,30	28,24	±	1,069	26,86	±	0,33
58	28,94	±	1,02	28,42	±	0,32	29,39	±	1,075	28,27	±	0,35
68	31,35	±	1,03	32,08	±	0,37	31,59	±	1,100	31,66	±	0,36
78	33,28	±	1,04	34,98	±	0,39	33,39	±	1,106	34,22	±	0,37
82	34,38	±	1,04	36,19	±	0,39	34,21	±	1,110	35,33	±	0,38
89	35,66	±	1,09	37,69	±	0,40	35,08	±	1,121	36,83	±	0,39
97	37,07	±	1,13	39,39	±	0,41	36,26	±	1,128	38,37	±	0,40
103	38,17	±	1,15	40,66	±	0,42	37,21	±	1,140	39,42	±	0,40
